# Supplementary material for: Surface properties of the seas of Titan as revealed by Cassini mission bistatic radar experiments
Source: Nat Commun. 2024 Jul 16;15:5454. doi: 10.1038/s41467-024-49837-2 (PMC11252143; doi:10.1038/s41467-024-49837-2)
Supplement: Supplementary file 1 — Supplementary Information [file 41467_2024_49837_MOESM1_ESM.pdf]

Supplementary Information

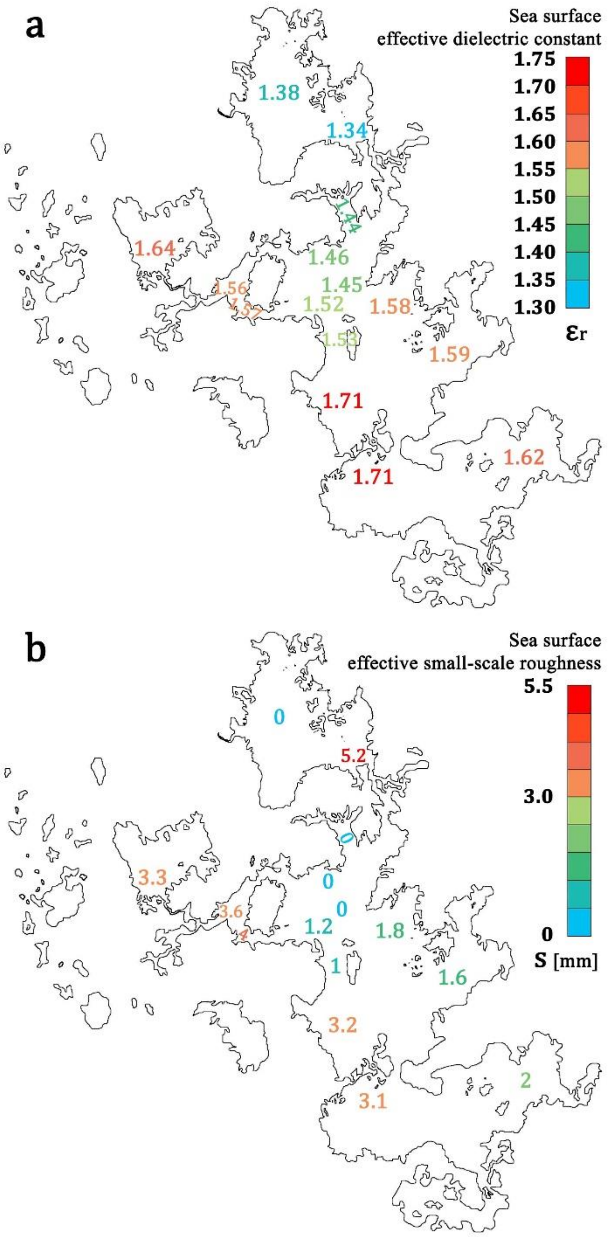

Figure S1. Simplified version of the polar stereographic projection reported in Figure 2 of this paper. *Here are represented the coastlines of Titan's three large polar liquid hydrocarbon seas and the results obtained for* ***(a)*** *the estimated effective dielectric constant for the areas of interest selected for this work;* ***(b)*** *the estimated small-scale roughness for the areas of interest selected for this work. Source data are provided as a Source Data file.*

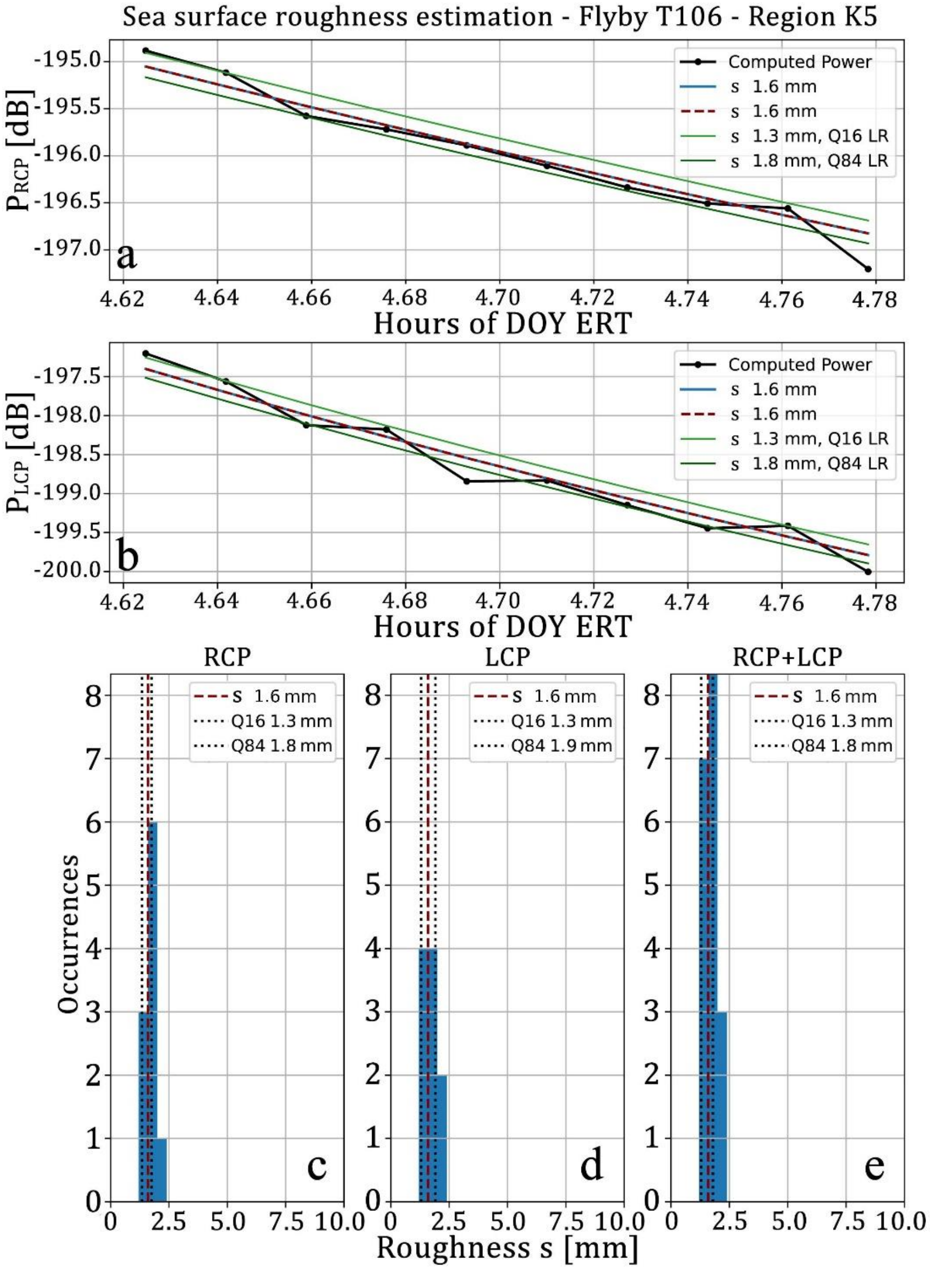

Figure S2. Example of sea surface roughness estimation*. In panels a and b, black curves indicate calibrated received powers for the left and right channels, blue and red curves show the best fitting model identified respectively on the right and left channel, the green lines indicate the* 1σ *errors. Panels c and d show the histograms obtained for the estimations performed respectively for the right and left channels separately. Panel e shows the histogram obtained by combining the estimations performed on the right and left channels. Source data are provided as a Source Data file.*
