# Supplementary material for: Surface properties of the seas of Titan as revealed by Cassini mission bistatic radar experiments
Source: Nat Commun. 2024 Jul 16;15:5454. doi: 10.1038/s41467-024-49837-2 (PMC11252143; doi:10.1038/s41467-024-49837-2)
Supplement: Supplementary file 3 — Description of Additional Supplementary Files [file 41467_2024_49837_MOESM3_ESM.docx]

**File Name:** Supplementary Software
**Description:** This is a rar archive containing some sample Python and Matlab codes useful to extract, process and calibrate the bistatic data acquired by the Cassini RSS instrument for the seas of Titan. More specifically, a Python code (rsr_reader.py) useful to extract the raw data provided by the RSS team (also publicly available on GitHub), a Matlab code useful to convert the raw data numbers of the I and Q channels in useful signal amplitudes on (RSS_BSR_READ_CVS.m), a Matlab code useful to understand how to process and calibrate the data (RSS_BSR_PROC.m). In the archive are present also four files carrying the ancillary information relative to the four flybys of interest for this work (bistatic_geometry_T101.mat, bistatic_geometry_T102.mat, bistatic_geometry_T106.mat, bistatic_geometry_T124.mat), a few documents descriptive of the T106 flyby observation (which is taken as an example to show what kind information should be retrieved in order to process any flyby) and finally a text file (notes.txt), which describes briefly the whole process of download, extraction, processing and calibration of the data.
